# Asymptomatic bilateral plantar nodules in an infant

**DOI:** 10.1016/j.jdcr.2026.08.034

**Published:** 2026-08-28

**Authors:** Dalia Alanazi, Mohammed Alahmadi, Renad Alfalah, Razan Alhussain, Salha Hakami

**Affiliations:** a Department of Dermatology, King Saud Medical City Hospital, Riyadh, Saudi Arabia; b Department of Dermatology, King Fahad General Hospital, Medina, Saudi Arabia; c Department of Pediatrics, Maternity and Children Hospital, Dammam, Saudi Arabia; d Department of Dermatology, King Salman Hospital, Riyadh, Saudi Arabia

**Keywords:** fibrolipomatous hamartoma, nodule, pedal papules, precalcaneal congenital fibrolipomatous hamartoma

## Case presentation

A healthy six-month-old infant presented with congenital bilateral plantar nodules that had remained stable in size over the course of 4 months. According to the mother, the lesions were first noticed when the patient was 2 months old. Initially, they were soft in consistency but had gradually become firmer over time. There was no history of trauma or pressure on the area. He was a full-term and otherwise healthy baby with no family history of similar lesions. On physical examination, bilateral, symmetrical, skin-colored nodules were present over the plantar heels (soles). The nodules were soft, compressible, and non-tender, with no overlying erythema, ulceration, or bleeding, purulent discharge, or any other cutaneous changes, as shown in Figs 1 and 2.

**Fig 1:**
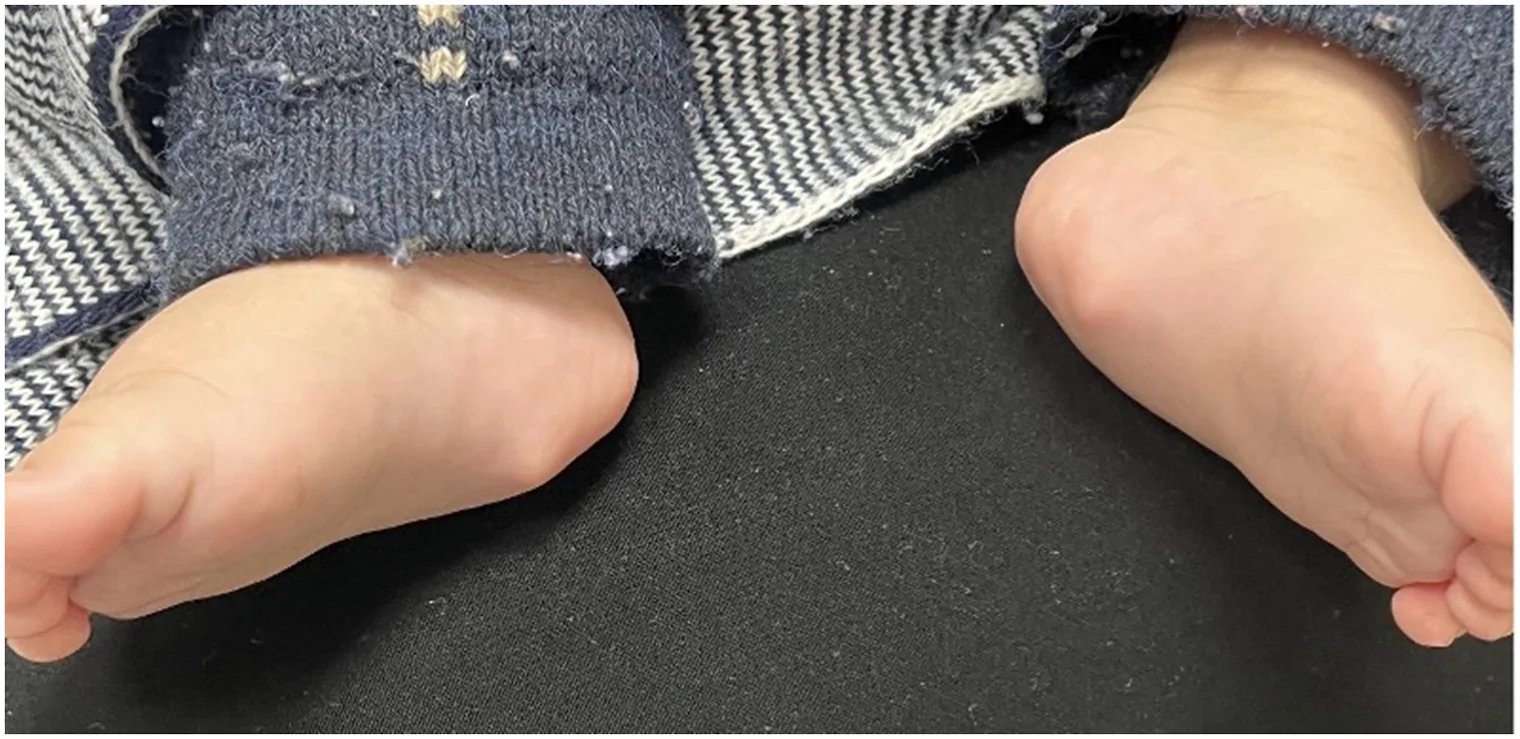
Bilateral skin colored subcutaneous rubbery nodules.

**Fig 2:**
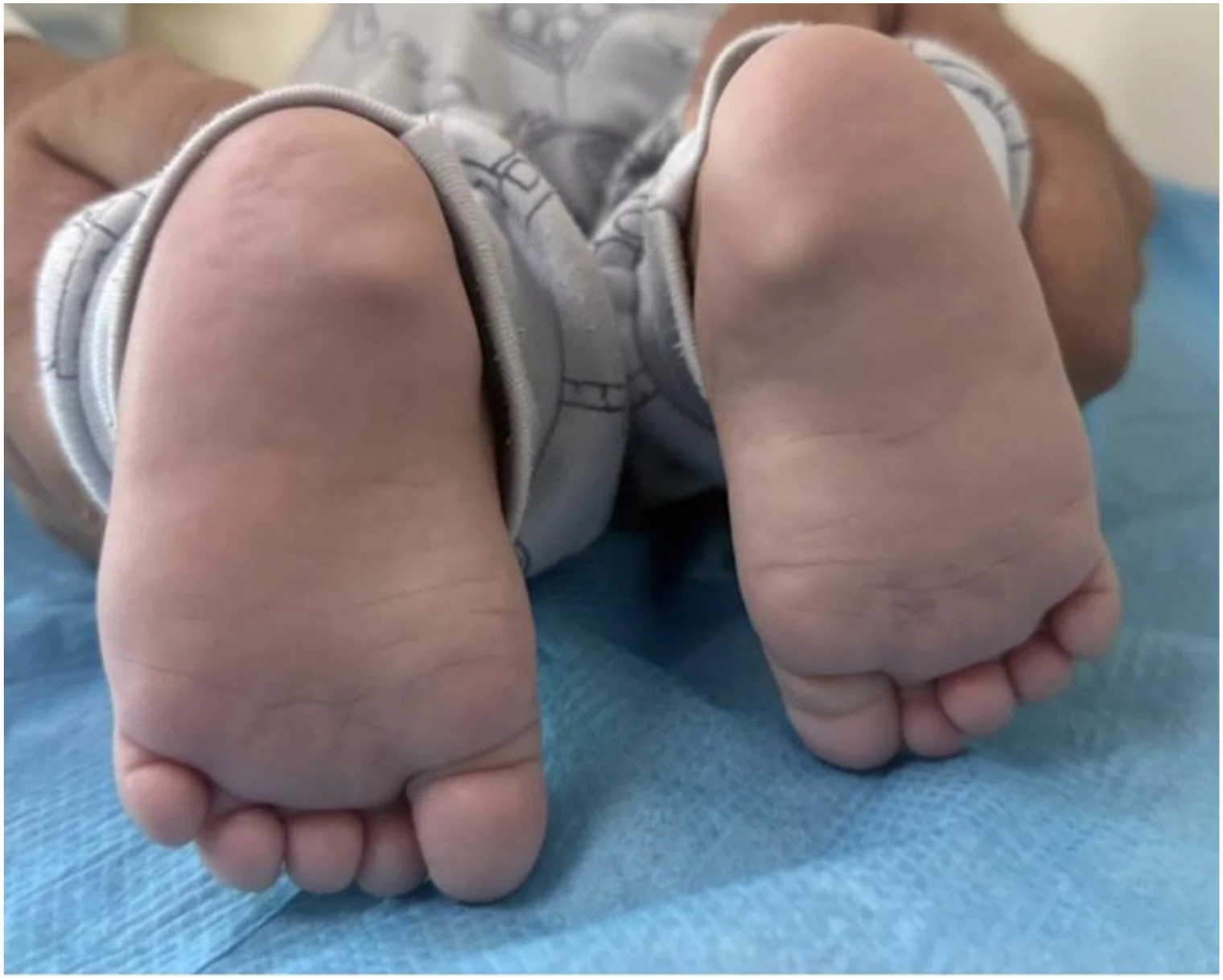
Bilateral skin colored subcutaneous rubbery nodules.

The diagnosis was established based on clinical examination only; no further tests were done. The mother was educated about the disease and its nature; no further treatment was provided, and she was reassured.

The clinical presentation was most consistent with precalcaneal congenital fibrolipomatous hamartomas (PCFHs). The differential diagnosis included infantile digital fibromatosis and solitary mastocytoma, although the latter was considered less likely given the bilateral presentation.

**Question: What is the preferred management of PCFH in most patients?**

**A.** Surgical excision
**B.** Intralesional corticosteroids
**C.** Observation and parental reassurance
**D.** Cryotherapy
**E.** Oral retinoids

**Correct answer:** C. Observation and parental reassurance.

## Discussion

PCFH, also known as bilateral congenital adipose plantar nodules, is an uncommon, benign lesion of infancy that typically presents at birth or during the first months of life as one or more asymptomatic, skin-colored, soft nodules located on one or both plantar heels. It has also been reported in the literature under multiple terms, such as pedal papules of the newborn, congenital fatty heel pads, and benign anteromedial plantar nodules. Although bilateral and symmetrical involvement is most characteristic, unilateral and atypical locations, including the retrocalcaneal, nasal, and mandibular regions, have also been reported. The lesions usually enlarge proportionally with the child’s growth or remain stable, without affecting gait or normal development, and there have been no reports of malignant transformation. Despite being considered rare, its true incidence may be underestimated because many cases are diagnosed clinically and remain unreported.^1^^,^^2^

The exact pathogenesis remains uncertain. Proposed mechanisms include persistence of fetal subcutaneous tissue due to incomplete regression during development, herniation of adipose tissue through defects in the plantar fascia, and a possible genetic basis with autosomal dominant or X-linked inheritance. However, most reported cases are sporadic, although familial clustering has been described.^2^^,^^3^

Diagnosis is primarily clinical. Ultrasonography is useful when the diagnosis is uncertain and typically demonstrates a homogeneous echogenic subcutaneous lesion with hypoechoic fibrous bands corresponding to mature adipose tissue separated by collagen fibers. Histopathological examination, which is rarely required, reveals mature adipocytes arranged in dermal lobules separated by collagenous fibrous septa beneath a normal epidermis. Since skin biopsy may be painful and unnecessary in infants, it should be reserved for atypical presentations or diagnostic uncertainty.^3^^,^^4^ The differential diagnosis includes juvenile fibromatosis, which is more indurated, unilateral, and appears in the second or third decade; piezogenic papules, which are pressure-based lesions; and calcified nodules, which are painful and due to repeated needle insertion in the heel.^1^^,^^5^

Management is conservative, consisting mainly of parental reassurance, as the condition is benign and usually asymptomatic. Most lesions remain stable or gradually regress during early childhood, although some may persist without causing functional impairment. Surgical intervention is rarely indicated and should be considered only for symptomatic or diagnostically uncertain cases.^1^, ^2^, ^3^, ^4^, ^5^

### Clinical importance

PCFH is likely under-recognized and under-reported because many clinicians are unfamiliar with its characteristic presentation. Failure to recognize this benign condition may result in unnecessary referrals, invasive diagnostic procedures, and increased parental anxiety. In most patients, the diagnosis can be established clinically, while histopathological examination should be reserved for atypical or diagnostically challenging cases. Further case reports and larger case series are needed to enhance understanding of its epidemiology, pathogenesis, and natural history.

### Declaration of generative AI and AI-assisted technologies in the writing process

None.

## Conflicts of interest

None disclosed.
